# Arsenical Melanodermia

**Published:** 1909-12-11

**Authors:** 


### Medicine.

### ARSENICAL MELANODERMIA.

### A CASE SIMULATING ADDISON'S DISEASE.

It is well enough known that various different skin lesions may result from the prolonged use of arsenic in medicinal doses. Amongst them, melanodermia is fairly frequent. Usually the melanodermia consists in the appearance of tawny or brownish spots, like freckles, scattered at wide intervals over the body. It is rarer to get generalised and continuous pigmentation, and cases of the latter may clearly be difficult of diagnosis.

### An Illustrative Case.

A good example of extensive melanodermia due to arsenic, but simulating Addison's disease, has been recorded by Enriquez and Lereboullet from the wards of Dr. Brissaud in Paris. The patient was a man aged 47, who had been perfectly well until one April, when he began to lose appetite and weight and to feel run down and generally out of sorts. He did not take any particular steps to remedy this until the following October, when he developed an eczematous eruption upon his limbs. He saw a medical man for this, and on October 19 he began to take a medicine containing Fowler's solution. As a rule he got sixteen drops of liquor arsenicalis a day—occasionally a little more. He persisted with this for months, neither increasing nor diminishing the dose. In January, three months after the beginning of the treatment, he noticed some prickling of his eyes, with a tendency to lachrymation, but there were no other signs of arsenical poisoning. It was not until the following March that the patient first noticed his skin going black. The pigmentation was at that time much more marked in some parts than in others, but it very quickly became general. By April or May it had extended over his entire body, and at the same time there was constant dryness of the throat and a tendency to lachrymation, especially on reading.

The medical man who was attending the patient at this time, relying upon the extreme degree of generalised pigmentation, which amounted in many places to veritable bronzing, upon progressive loss of flesh, and upon some degree of asthenia, thought he was suffering from Addison's disease, and ordered the Fowler's solution to be continued. Dr. Brissaud was of the same opinion when he saw the case a little later, ordering suprarenal extract in addition to the arsenic.

From that time the symptoms became more and more marked. The melanodermia increased, and there was continued loss of weight which might have been serious had the patient not been decidedly stout to start with. Gastro-intestinal symptoms, on the other hand, were conspicuous by their absence. There had been no lumbar aches; at most there had been one or two bouts of slight abdominal pain resembling mild biliary colic.

Asthenia, almost hypothetical from the beginning, did not increase, and the man continued to work hard. A year later Dr. Brissaud, struck by this remarkable absence of asthenia, took him into hospital for closer observation. He was a big strong man, naturally dark, but now absolutely bronzed as to the face. On stripping him the generalised melanodermia was most remarkable. The coloration was universal, and yet it was far from being equally deep everywhere. Tawny at some places, bronze-like in others, in some parts it was almost black. Addison's disease was undoubtedly one's first impression.

On closer inspection it could be noted that, although there was excess of pigment everywhere, there were numbers of paler areas or spots the size of peas or lentils. These were most numerous upon the thorax and abdomen, but they could also be made out upon the limbs. Conversely, there were other small areas in which the pigmentation was most intense, recalling the speckles that are to be seen in certain sorts of tobacco. These particularly dark spots were mostly upon the neck, behind the ears, and in front of and behind the axillæ. Roughly speaking, all flexures were pigmented with special intensity. The face was uniformly and relatively speaking slightly coloured; and it was remarkable how the pigmentation left the hands and the feet almost completely alone.

The bucco-labial mucous membrane was hardly affected; but there was one definite patch of pigment, the area of a lentil, and dark purplish brown, upon the inner aspect of the right half of the lower lip; moreover, the two labial commissures, especially the right, were slightly but distinctly pigmented. There was no pigmentary deposit to be detected upon the inner aspect of either cheek.

### Some Conclusions.

There was not a trace of any peripheral neuritis, and it might be urged that perhaps the case was really one of Addison's disease after all. Not only, however, was there a complete absence of all the other symptoms of Addison's disease, but also in confirmation of the diagnosis of arsenical pigmentation was the fact that the palms of the hands and the soles of the feet were in a state of typical hyperkeratosis. The skin of the palm of each hand was extremely thick and tough, with numerous small hard painless papules the size of small peas projecting from it; the palmar surfaces of the digits were less affected, and their dorsal surfaces not at all. On the feet the hyperkeratosis was still more marked, and there could be no doubt as its being due to the arsenic. It is particularly to be noted that the extreme degree of pigmentation in this case was due, not to any excessive use of arsenic, but to its continuance in medicinal doses over a long period.

